# Oral nutritional supplements for preventing surgical site infections: protocol for a systematic review and meta-analysis

**DOI:** 10.1186/s13643-020-01293-x

**Published:** 2020-02-20

**Authors:** Nicholas Ralph, Lindsay Brown, Kristy L. McKillop, Jed Duff, Sonya Osborne, Victoria R. Terry, Karen-Leigh Edward, Rachel King, Edward Barui

**Affiliations:** 1 School of Nursing and Midwifery, University of Southern Queensland, Toowoomba, Australia; 2 Division of Research and Innovation, University of Southern Queensland, Toowoomba, Australia; 3 Faculty of Health, University of Technology Sydney, Ultimo, Australia; 4 School of Health and Wellbeing, University of Southern Queensland, Toowoomba, Australia; 5 St Vincent’s Private Hospital, Toowoomba, Australia; 6 School of Nursing and Midwifery, The University of Newcastle, Callaghan, Australia; 7 School of Public Health and Social Work, Institute of Health and Biomedical Innovation, Queensland University of Technology, Brisbane, Australia; 8 School of Nursing, Swinburne University, Melbourne, Australia; 9 School of Sciences, University of Southern Queensland, Toowoomba, Australia

**Keywords:** Surgical site infection, Malnutrition, Nutrition, Surgery

## Abstract

**Background:**

Surgical site infections (SSIs) are among the most common healthcare-associated infections. Under-nutrition is an important risk factor for SSIs and can lead to delayed wound healing and longer hospital stays. Oral nutritional supplements are prescribed to reduce the risk of infection and improve health status, but data from randomised controlled trials (RCTs) have shown mixed results. Thus, the objective of our planned systematic review is to evaluate oral nutritional supplements on preventing SSIs in adult surgical patients

**Methods:**

RCTs conducted in adult surgical patients who receive oral nutritional support will be included. The primary outcome will be the incidence of SSIs (within 30 days of surgery or within 90 days for joint replacement surgery). Secondary outcomes will be changes in nutritional status, mortality, health-related quality of life and costs. Literature searches will be conducted in several electronic databases (from inception onwards): MEDLINE, Embase, CINAHL and The Cochrane Central Register of Controlled Trials (CENTRAL). Grey literature will be identified through searching clinical trial registers and dissertation databases. Two reviewers will independently screen all citations, full-text articles and abstract data. The study methodological quality (or bias) will be appraised using the Cochrane risk of bias tool. If feasible, we will conduct random effects meta-analysis where appropriate.

**Discussion:**

This systematic review will evaluate the evidence for pre- and post-surgical intervention with oral nutritional supplements in adults. Findings from this planned review may inform subsequent nutritional interventions for hospitalised patients who undergo surgery.

**Systematic review registration:**

PROSPERO CRD42020140954

## Background

Surgical site infections (SSIs) are defined by the Centers for Disease Control and Prevention (CDC) as infections that occur after surgery in the part of the body or the incision where the surgery took place. SSIs range from simple wound infections involving the skin and subcutaneous tissues (classified as superficial incisional), to deep soft-tissue infections involving fascia and muscle (classified as deep incisional), or infections of the space or organ manipulated during the surgical procedure (classified as organ/space) [1]. SSIs are among the most common healthcare-associated infections, as the World Health Organisation (WHO) estimates a pooled global SSI incidence rate of 11.2% [2]. However, variations of SSI incidence are marked. In one North American study of over 750,000 surgical patients, 1% of procedures resulted in an SSI, with similar incidence rates found in other developed health systems [3, 4]. In a study of 75,695 patients in hospitals in the United Kingdom (UK) over a 4-month period, 8% of patients suffered a healthcare-associated infection, with SSIs making up 15% of these infections [5]. Depending on whether the surgery is classified as clean, clean/contaminated, contaminated or dirty, SSIs can also occur at different rates [6]. Nonetheless, the prevalence of SSIs may be under-estimated as most present within 30 days following a surgical procedure; a proportion of which is likely to develop outside of hospital [7, 8].

SSIs result in delayed wound healing, increased hospital stays, increased use of antibiotics, unnecessary pain and, in extreme cases, death of the patient [9]. SSIs result in delayed wound healing, increased hospital stays, increased use of antibiotics, unnecessary pain, death of the patient in extreme cases, as well as increased health resource use and expenditure depending of the location, depth and severity of the infection [10, 11]. Factors that contribute to the development of SSI include the patient’s health status, the type of surgery conducted and the physical environment where surgical care is provided [12]. Surgical risk factors include emergency surgery or surgeries involving major blood loss, surgery involving contaminated or dirty wounds and prolonged surgery [9]. Patient-level factors include a preoperative American Society of Anesthesiology (ASA) score of III or IV [13, 14], high body mass index (BMI) > 35, malnutrition, advanced age and the pre-existence of immune-compromising conditions or diseases such as diabetes and cancer and malnutrition from inadequate nutritional intake [15, 16]. Hospitalisation is also associated with a deterioration of nutritional intake in admitted elective surgical patients leading to under-nutrition [17].

Malnutrition can cause poor clinical outcomes from surgery through disruption to physiological and psychological health [18]. Although no universally accepted definition is clear, malnutrition can be broadly described as any imbalance in an individual’s nutritional status which affects body composition and/or function: such imbalance might relate to overnutrition through excessive nutritional intake or under-nutrition caused by insufficient nutritional intake or malabsorption [19–21]. Most screening tools typically only distinguish undernourished patients rather than stratifying nutritional status according to whether they are of normal weight or obese/overweight [22, 23]. Consequently, the stratification of risk among malnourished, normal weight and obese patients is poorly understood. Moreover, compliance with routine screening of nutritional status is inconsistently performed in hospitals, even in countries such as the UK and the United States of America (USA) where it is mandatory on admission [24, 25]. Although the quality of research varies, reported under-nutrition ranges from 30 to 55% in hospitalised patients in studies conducted across a range of countries [26–30].

Oral nutritional supplements contain either macronutrients (proteins, fats, carbohydrates and amino acids) and/or micronutrients (vitamins and minerals) to supplement the oral diet. Some ingredients in oral nutritional supplements such as proteins, arginine, glutamine, omega-3 fatty acids, vitamins and trace minerals may improve immune function and wound healing [31]. Nutrients such as arginine and glutamine are typically referred to as immunonutrition. Oral nutritional supplements containing macronutrients, micronutrients or a mixture of both are usually prescribed if under-nutrition is diagnosed or the patient is assessed as being nutritionally at-risk, and an improvement in the nutritional status is desired or required [20, 32]. To achieve improvements in nutritional status, oral nutritional supplements are usually prescribed for at least 7 days before surgery [33] and up to 4 weeks postoperatively [34]; however, protocols vary. Typically, oral nutritional supplements are taken as liquids up to three times a day with an intake of about 250–600 kcal/daily usually in addition to daily dietary intake [18].

Under-nutrition results from a shortfall between nutrition intake and nutrient requirements leading to tissue loss and changes to normal physiological function [35]. Surgical patients may be undernourished on admission to hospitals [36]. Surgery can exacerbate under-nutrition by causing a systemic inflammatory response which increases metabolic activity, elevates energy consumption, impairs organ function and compromises immunity [37, 38]. Undernourished patients may be at risk of developing SSI [15, 39], are at a greater risk of death and morbidity [40, 41] and require more hospital resources than normally nourished patients [42, 43]. Oral nutritional supplements containing protein and/or arginine may work to improve nutrition status and reduce the risk of developing an SSI [44–48].

Findings reported from RCTs of oral nutritional supplementation have been mixed across studies of both single and multi-nutrient oral nutritional supplements. For studies investigating the effect of single-nutrient oral nutritional supplementation with a formula of either protein, arginine or amino acids, a reduced rate of postoperative infection complications was reported among head and neck cancer surgery patients but the effect was not statistically significant [49, 50]. Two further studies were conducted to measure the effect of single-nutrient oral nutritional supplements with no SSIs recorded across the intervention control groups of two studies [44, 45]. One study reported a statistically insignificant increase in SSIs following single-nutrient oral nutritional supplementation and compared with the control group [51].

Similarly, mixed findings have been reported from RCTs of multi-nutrient oral nutritional supplements defined as a formula of two or more nutrients comprising energy sources and protein, arginine or amino acids. In two studies, fewer SSIs were reported among individuals who received multi-nutrient supplements across two RCTs than those who received routine nutrition [46–48]. In similar studies of multi-nutrient oral nutritional supplements, no statistically significant difference in SSI rates was demonstrated [52–54] whilst one study reported that multi-nutrient oral nutritional supplements may increase SSIs relative to routine nutrition [55].

To understand the available evidence and its quality, it is important to search, analyse and report on the role of oral nutritional supplements for preventing SSIs. This systematic review may provide evidence to inform clinical practice, as well as highlighting further areas for investigation. Since SSIs have marked negative effects on the person, health services and society across the globe [56], it is important to identify effective interventions to reduce their incidence. Principally, this review is necessary to help find, appraise and summarise the current evidence on the advantages and disadvantages of oral nutritional supplements and present findings accordingly. Thus, the objective of our planned systematic review is to evaluate oral nutritional supplements on preventing SSIs in adult surgical patients.

## Methods/design

### Inclusion and exclusion criteria

We will include published RCTs that include a statement of peer review including theses, cluster RCTs and N of 1 trials. We will include unpublished trials located from conference abstracts and proceedings. We will only include studies written in English as we do not have access to scientific translation services. Studies using quasi-randomisation will be excluded. We will include studies of adults 18 or over of any gender who undergo surgery, with or without abnormal body composition, for example, any BMI, of any nutritional status or disease state. We will not exclude patients by surgical type or by hospital setting. However, it is likely that most studies will investigate patients undergoing major surgery such as joint replacement, as oral nutritional supplements are not typically prescribed for individuals undergoing minor surgery such as cataract surgery.

We will include RCTs recruiting people described in the primary report as receiving oral nutritional supplements or no oral nutritional supplements or a placebo in the hospital setting who undergo a surgical procedure. Although we expect most studies will focus on oral nutritional supplements as a treatment for under-nutrition, we will include studies where oral nutritional supplements are prescribed to supplement the diet of normally nourished or obese patients. As the method of assessing and defining nutritional status may vary, we will accept definitions of normal nutrition and malnutrition as used by the study authors.

The primary intervention of interest is oral nutritional supplements containing either macronutrients, micronutrients or a mixture of both. We will exclude studies investigating supplements that do not contain an energy source (carbohydrates, fats, proteins). We will also exclude studies of single-nutrient oral nutritional supplements or parenteral nutrition. We will include RCTs in which the use of oral nutritional supplements during the treatment period, either preoperatively or postoperatively, is the only interventional difference between treatment groups. We anticipate that likely comparisons will include protein-enriched supplements compared with immunonutrition supplements, such as supplements enriched with arginine or glutamine, used during the care pathway and added to standard practice, comparisons of different types/brands of oral nutritional supplements or comparisons of oral nutritional supplements with a placebo or control such as no supplementation. The duration and frequency of oral nutritional supplements compared with a placebo or control will be reported.

We will exclude prebiotics, probiotics and synbiotics as they are ingredients in food that are undigested in the stomach but metabolised in the colon to promote bacterial growth or activity which may benefit health [57]. We will also exclude products which are administered for purposes other than improving nutritional status. As an example, medicinal herbs can be derived from edible foods such as ginger, garlic, dandelion, lavender, fennel, thyme, mint, liquorice, chamomile and St John’s Wort and can be taken to achieve a pharmaceutical or pharmacokinetic effect. All medicinal herbs, herbal therapies, homoeopathic substances and zinc supplements will be excluded from the review. We will only review interventions where oral nutritional supplements have been taken to supplement the oral diet as this is a typical approach for targeting improvements in the nutritional status of surgical patients in the hospital setting as well as the phenomena of interest in this review. Patients on total parenteral nutrition (TPN) or receiving supplements via a nasogastric tube will be excluded from the review.

### Outcome measures

We list primary and secondary outcomes below. If a study is otherwise eligible (i.e. correct study design, population and intervention/comparator) but does not report a listed outcome, then we will contact the study authors where possible to establish whether an outcome of interest here was measured but not reported. Studies will be excluded where it is clear that our primary outcome was not measured.

We will report outcome measures at the latest time point available, assumed to be length of follow-up if not specified, and the time point specified in the methods as being of primary interest, if this is different from the latest time point available. For all outcomes, we will class assessment of outcome measures in 2 categories:

- From up to 30 days after surgery for non-joint replacement surgery
- From up to 90 days after surgery for joint-replacement surgery

The primary outcome of this review is an evaluation of oral nutrition supplements in SSIs. In the latest guidelines for prevention of SSIs [1], the CDC defines SSIs as ‘infections of the incision or organ or space that occur after surgery’ (p785) and standardises the types of SSIs into (a) superficial incisional, (b) deep incisional and (c) organ space (CDC 2018b). Despite this, diagnosis of SSIs varies between studies.

We will, therefore, accept the definition used by the original authors to determine:

- The proportion of patients who developed any SSIs before or after discharge from hospital within 30 days following surgery.
- For joint replacement surgery, we will limit reporting to infections which occur within the first 90 days following surgery as per CDC diagnostic guidelines which include this period in the SSI risk period [58].
- We will report any reported incident of SSIs that falls under these definitions as 1 event.

Secondary outcomes are:

- Changes to nutritional status. We will report biological and immunological marker changes compared with intervention and the control groups including total protein (g/L), albumin (g/dl), prealbumin (mg/dl) and total lymphocytes (10^3^ in 1 μL/mm^3^ of blood). We will accept any validated nutritional assessment tool measures taken at least once prior to supplementation and at least once after supplementation is ceased;
- Costs. Any cost effectiveness that relates costs to benefits including but not limited to cost-utility or incremental cost-effectiveness ratio;
- Mortality. We will include death up to 52 weeks following surgery; and
- Health-related quality of life. We will include health-related quality of life when reported where a validated scale such as the SF-36 or EQ-5D was used. We will not report ad hoc measures of quality of life that are unlikely to be common to trials and unvalidated.

### Search strategy

We will search the following electronic databases to retrieve reports of relevant randomised clinical trials: the Cochrane Wounds Group Specialised Register (to present), the Cochrane Central Register of Controlled Trials (CENTRAL) (the Cochrane Library, latest issue), Ovid MEDLINE (1946 to present), Ovid MEDLINE (In-Process & Other Non-Indexed Citations), Ovid Embase (1974 to present) and EBSCO CINAHL Plus (1937 to present).

We will use the provisional search strategy in Additional file 1 to search the Cochrane Central Register of Controlled Trials (CENTRAL). We will combine the Ovid MEDLINE search with the Cochrane Highly Sensitive Search Strategy for identifying randomised trials in MEDLINE: sensitivity- and precision-maximising version (2008 revision) [59]. We will combine the Embase search with the Ovid Embase filter developed by the UK Cochrane Centre [59]. We will combine the CINAHL Plus search with the trial filters developed by the Scottish Intercollegiate Guidelines Network [60]. There will be no restrictions with respect to date of publication or study setting. We will also search the following clinical trial registries: ClinicalTrials.gov, WHO International Clinical Trials Registry Platform and EU Clinical Trials Register.

We will contact the corresponding authors for study information and the manufacturers and distributors of nutritional supplements for product information where this is required. We will try to identify other potentially eligible trials or ancillary publications by searching the reference lists of retrieved included trials as well as relevant systematic reviews, meta-analyses and health technology assessment reports.

### Screening

Two review authors will independently assess the titles and abstracts of the citations retrieved by the searches for relevance. After this initial assessment, we will obtain full text copies of all studies considered to be potentially relevant. In teams of two, review authors will independently check the full papers for eligibility; disagreements will be resolved by discussion and, where required, the input of a third review author. Where required and possible, we will contact study authors where the eligibility of a study is unclear. We will record all reasons for exclusion of studies for which we had obtained full copies. We will complete a PRISMA flowchart to summarise this process and a PRISMA-P checklist is also appended (see Additional file 2) [61]. Where studies have been reported in multiple publications/reports, we will obtain all publications. Whilst the study will be included only once in the review, data will be extracted from all reports to ensure maximal relevant data are obtained.

### Extraction

We will extract and summarise details of the eligible studies using a standardised data extraction sheet. Review authors will extract data independently in pairs and will resolve disagreements by discussion, drawing on a third review author where required. Where data are missing from reports, we will attempt to contact the study authors to obtain this information. Where a study with more than two intervention arms is included, only data from intervention and control groups that meet the eligibility criteria will be extracted.

We will extract the following data where possible by treatment group for the pre-specified interventions and outcomes in this review. Outcome data will be collected for relevant time points including:

- Country of origin
- Type of wound and surgery
- Unit of randomisation (per patient)—single wound or multiple wounds on the same patient
- Unit of analysis
- Trial design such as parallel, cluster
- Care setting
- Number of participants randomised to each trial arm
- Eligibility criteria and key baseline participant data
- Details of treatment regimen received by each group
- Commencement, end and duration of treatment
- Details of any co-interventions
- Primary and secondary outcome(s) (with definitions)
- Outcome data for primary and secondary outcomes (by group)
- Duration of follow-up
- Number of withdrawals (by group)
- Blinding (both patient and professional)
- Publication status of study
- Source of funding for trial
- Patient demographics such as gender and age

### Quality assessment

Two review authors will independently assess included studies using the Cochrane tool for assessing risk of bias [62]. This tool addresses six specific domains: sequence generation, allocation concealment, blinding, incomplete data, selective outcome reporting and other issues. In this review we will record issues with unit of analysis, for example, where a cluster trial has been undertaken but analysed at the individual level in the study report. We will assess blinding and completeness of outcome data for each of the review outcomes separately. We note that, since wound healing is a subjective outcome, it can be at high risk of measurement bias when outcome assessment is not blinded. We will present our assessment of risk of bias using two ‘risk of bias’ summary figures; one which is a summary of bias for each item across all studies, and a second which shows a cross-tabulation of each trial by all of the ‘risk of bias’ items. We will class studies with an assessment of high risk of bias for the randomisation sequence domain and/or the allocation concealment domain and/or the blinded outcome assessment domain (for specified outcome) as being at overall high risk of bias (for specified outcome). Where risk of bias judgement is made based on information from correspondence with trial authors, this will be noted in the risk of bias table. For trials using cluster randomisation, we will also consider the risk of bias in terms of recruitment bias, baseline imbalance, loss of clusters, incorrect analysis and comparability with individually randomised trials [63].

### Analysis

For dichotomous outcomes, we will calculate the odds ratio (OR) with 95% confidence intervals (CI). For continuously distributed outcome data, we will use the difference in means (MD) with 95% CIs, if all trials use the same or similar assessment scale. If trials use different assessment scales, we will use the standardised mean difference (SMD) with 95% CIs. We will only consider mean or median time to healing without survival analysis as a valid outcome if reports specify that all wounds healed, meaning if the trial authors regarded time to healing as a continuous measure as there is no censoring. Time-to-event data such as time-to-complete wound healing will be reported as hazard ratios (HR) where possible in accordance with the methods described in the Cochrane Handbook [64]. If studies reporting time-to-event data such as time to healing do not report a hazard ratio, then, where feasible, we plan to estimate this using other reported outcomes, such as the numbers of events, through the application of available statistical methods [65]. We will use the DerSimonian-Laird method to combine OR as we anticipate using a random effects model due to likely study heterogeneity [65].

Where studies randomise at the participant level and measure outcomes at the wound level, such as wound healing, we will treat the participant as the unit of analysis when the number of wounds assessed appears equal to the number of participants (e.g. one wound per person). There may be instances of clustered data where a proportion of individually randomised trial participants have outcome data collected and reported on multiple wounds. Since not all participants will have multiple wounds, this is not a cluster trial per se but rather a trial that incorrectly includes a mixture of individual and clustered data. Such trials will be noted and the issue will be recorded in the risk of bias assessment. Data will be extracted and presented but will not be the subject of any further analyses.

We will only incorporate clearly conducted fully cluster trials into meta-analyses if the trial has been analysed correctly. Where a cluster trial has been conducted but incorrectly analysed we will record this as part of the ‘risk of bias’ assessment. If possible, we will approximate the correct analyses based on Cochrane Handbook guidance [63] using information on the number of clusters (or groups) randomised to each intervention group or the average (mean) size of each cluster; the outcome data ignoring the cluster design for the total number of individuals (for example, number or proportion of individuals with events, or means and standard deviations) and an estimate of the intracluster (or intraclass) correlation coefficient (ICC). Where multiple trial arms are reported in a single trial, we will include only the relevant arms. If two interventions or more interventions are compared with the control and are eligible for the same meta-analysis, we will pool the intervention arms and compare that with the control. If the study data cannot be analysed correctly, outcome data will be extracted and presented but not analysed further.

It is common to have data missing from trial reports. Excluding participants’ post-randomisation from the analysis, or ignoring those participants who are lost to follow up, compromises the randomisation and potentially introduces bias into the trial. Where there are missing data we think should be included in the analyses, we will contact the relevant study authors to request whether these data are available. We will adopt the process detailed by Miller to evaluate the potential bias of bivariate and multivariate comparisons of ‘stayers and leavers’ [66]. For all secondary outcomes, we will present available data from the study reports/study authors and do not plan to impute missing data. Where measures of variance are missing, we will calculate these wherever possible. If calculation is not possible, we will contact the study authors. Where these measures of variance are not available, we will exclude the study from any relevant meta-analyses that are conducted and discuss the potential implications of its absence from a meta-analysis.

### Assessment of heterogeneity

Assessment of heterogeneity can be a complex, multi-faceted process. Firstly, we will consider clinical and methodological heterogeneity, that is, the degree to which the included studies vary in terms of participant, intervention, outcome and characteristics such as length of follow-up. This assessment of clinical and methodological heterogeneity will be supplemented by information regarding statistical heterogeneity, assessed using the chi-squared test where a significance level of *P* < 0.10 will be considered to indicate statistically significant heterogeneity, in conjunction with the *I*^2^ measure [67]. *I*^2^ examines the percentage of total variation across RCTs that is due to heterogeneity rather than chance [67]. In general, *I*^2^ values of 40%, or less, may not be important [67]; and values of more than 75%, or more, may indicate considerable heterogeneity [64]. We will attempt to explore further where there is evidence of high heterogeneity.

### Assessment of bias

Reporting biases arise when the dissemination of research findings is influenced by the nature and direction of results. Publication bias is one of a number of possible causes of small study effects, that is, a tendency for estimates of the intervention effect to be more beneficial in smaller RCTs. We will produce funnel plots as a visual assessment of whether small study effects may be present in a meta-analysis and as a means of estimating the intervention effects from individual RCT against some measure of each trial’s size or precision [68]. We plan to present funnel plots for meta-analyses comprising 10 RCTs or more.

### Outcomes

Details of included studies will be combined in a narrative review according to type of comparator, possibly by location/type of wound and then by outcomes by time period. Clinical and methodological heterogeneity will be considered and pooling undertaken when studies appear appropriately similar in terms of wound type, intervention type, duration of follow-up and outcome type.

We are unable to pre-specify the amount of clinical, methodological and statistical heterogeneity in the included studies but it might be extensive. Thus, we anticipate using a random effects approach for meta-analysis. Conducting meta-analysis with a fixed effect model in the presence of even minor heterogeneity may provide overly narrow confidence intervals. We will only use a fixed effect approach when clinical and methodological heterogeneity are assessed to be minimal, and the assumption that a single underlying treatment effect is being estimated holds. Chi-squared and *I*^2^ will be used to quantify heterogeneity but will not be used to guide choice of model for meta-analysis. We will exercise caution when meta-analysed data are at risk of small study effects because a random effects model may be unsuitable. In this case, or where there are other reasons to question the selection of a fixed effect or random effects model, we will assess the impact of the approach using sensitivity analyses to compare results from alternate models. We will report any evidence that suggests that the use of a particular model might not be robust. We may meta-analyse except when there is thought to be extensive heterogeneity. We will attempt to explore the causes behind this using meta-regression, if possible [69].

Data will be presented using forest plots where possible. For dichotomous outcomes, we will present the summary estimate as an odds ratio (OR) with 95% CI. Where continuous outcomes are measured in the same way across studies, we plan to present a pooled MD with 95% CI; we plan to pool [70] SMD estimates where studies measure the same outcome using different methods. For time-to-event data, we plan to plot and, if appropriate, to pool, estimates of hazard ratios and 95% CIs as presented in the study reports using the generic inverse variance method. Where time to healing is analysed as a continuous measure but it is not clear if all wounds healed, use of the outcome in the study will be documented but data will not be summarised or used in any meta-analysis.

We will present the main results of the review in ‘summary of findings’ tables; one table per comparison. The tables will contain key information concerning the quality of the evidence, the magnitude of the effects of the interventions examined and the sum of the available data for the main outcomes [71]. The ‘summary of findings’ tables will also include an overall grading of the evidence related to each of the main outcomes using the GRADE (Grades of Recommendation, Assessment, Development and Evaluation) approach [72]. The GRADE approach defines the quality of a body of evidence as the extent to which one can be confident that an estimate of effect or association is close to the true effect. The quality of a body of evidence involves consideration of within-trial risk of bias (methodological quality), inconsistency between studies (heterogeneity), directness of evidence (generalisability of population or outcomes), precision of effect estimates and risk of publication bias [70]. We will downgrade evidence using an approach adapted from Dijkers (see Table 1) [73].

**Table 1:** Factors that may lead to downgrading or upgrading of evidence in the GRADE approach

Criteria for downgrading	By how many levels
1. Serious risk of bias	Serious (- 1) or very serious (- 2) limitation to study quality
2. Serious inconsistence between studies	Important inconsistency, lack of agreement between studies or conflicting results (- 1)
3. Serious indirectness	Uncertainty with generalisability of population or outcomes (- 1) or with both population and outcomes (- 2)
4. Serious imprecision	Imprecise or sparse data (- 1)
5. Likely publication bias	High probability of reporting bias (- 1)

We plan to present the following outcomes in the ‘summary of findings’ tables: SSIs incidence, changes to nutritional status, cost, mortality and health-related quality of life. If sufficient data are available, we will carry out subgroup analyses in order to determine potentially important differences in the characteristics of interventions or the effect of interventions on different groups. We will assess potential heterogeneity. We will consider the following subgroups:

1. Age such as < 18 years, ≥ 18 years and < 65 years and ≥ 65 years
2. Gender
3. Type of surgery such as clean, clean/contaminated, contaminated or dirty
4. Body composition according to CDC [74] definitions of underweight, normal weight and overweight such as BMI > 30 obese
5. Type of intervention such as protein-based or arginine-based or both [75]
6. Degree of nutrition status such as classifications of under-nutrition over nutrition or normal nutrition as assessed by any nutrition assessment tool or as defined by study authors [19–21]
7. Treatment regimen of nutrition supplement such as time of initiation and period of dosing [76]
8. Low risk of bias versus unclear or high risk of bias
9. Biomarker levels (normal versus abnormal ranges)

### Sensitivity analyses

Where possible, we plan to perform sensitivity analyses to explore the impact or influence of key assumptions or variations on effect estimates related to different nutritional assessment tools used to define nutrition status.

## Discussion

Since evaluating nutritional status, complications and interventions are complex; we anticipate there may be few studies that appropriately address the research question. Therefore, we will conduct preliminary searches to scope the literature and systematically refine the search to ensure inclusion of all relevant literature. Additionally, searching and retrieving theses may prove to be complex. However, we will use ProQuest Theses and Dissertation database as well as the Gray Matters Application to minimise the chance of missing potential studies. Given heterogeneity between interventions, outcomes and research designs is likely, it may not be appropriate to meta-analyse the studies. Also, without large, cluster randomised controlled trials distinguishing interventional effectiveness across surgical demographics will be challenging. We will perform an analysis of evidence quality to counter this potential gap.

## Supplementary information

**Additional file 1.** Provisional search strategy

**Additional file 2.** PRISMA-P 2015 Checklist

## Data Availability

Not applicable

## Abbreviations

ASA: American Society of Anesthesiology
BMI: Body mass index
CDC: Centers for Disease Control and Prevention
CI: Confidence intervals
GRADE: Grades of Recommendation, Assessment, Development and Evaluation
HR: Hazard ratios
ICC: Intracluster (or intraclass) correlation coefficient
MD: Difference in the means
OR: Odds ratio
RCT: Randomised controlled trial
SMD: Standardised mean difference
SSI: Surgical site infection
TPN: Total parenteral nutrition
UK: United Kingdom
USA: United States of America
WHO: World Health Organisation
