# Supplementary material for: Oral nutritional supplements for preventing surgical site infections: protocol for a systematic review and meta-analysis
Source: Syst Rev. 2020 Feb 20;9:37. doi: 10.1186/s13643-020-01293-x (PMC7031994; doi:10.1186/s13643-020-01293-x)
Supplement: Supplementary file 1 — Additional file 1. Provisional search strategy [file 13643_2020_1293_MOESM1_ESM.docx]

**Additional file 1**

**Provisional search strategy**

#1 MeSH descriptor: [Dietary Supplements] explode all trees
#2 MeSH descriptor: [Micronutrients] explode all trees
#3 MeSH descriptor: [Dietary Proteins] explode all trees
#4 MeSH descriptor: [Dietary Carbohydrates] explode all trees
#5 MeSH descriptor: [Dietary Fats] explode all trees
#6 MeSH descriptor: [Energy Intake] explode all trees
#7 (diet* near/3 (supplement* or fortification or capsule* or tablet* or liquid*)):ti,ab,kw
#8 (nutrient* near/3 (supplement* or fortification or capsule* or tablet* or liquid*)):ti,ab,kw
#9 ((micronutrient* or micro-nutrient* or vitamin* or multivitamin* or mineral* or trace next element* or zinc or iodine or iron or cobalt or chromium or copper or manganese or fluoride or sodium or selenium or molybdenum) near/3 (supplement* or fortification or capsule* or tablet* or liquid*)):ti,ab,kw
#10 ((macronutrient* or macro-nutrient* or protein* or amino next acid* or carbohydrate* or calorie* or energ* or fat* or lipid*) near/3 (supplement* or fortification or capsule* or tablet* or liquid*)):ti,ab,kw
#11 ((food or diet) near/3 (intake or fortif*)):ti,ab,kw
#12 (or #1-#11)
#13 MeSH descriptor: [Surgical Wound Infection] explode all trees
#14 MeSH descriptor: [Surgical Wound Dehiscence] explode all trees
#15 (surg* near/5 infect*):ti,ab,kw
#16 (surg* near/5 wound*):ti,ab,kw
#17 (surg* near/5 site*):ti,ab,kw
#18 (surg* near/5 incision*):ti,ab,kw
#19 (surg* near/5 dehisc*):ti,ab,kw
#20 (wound* near/5 dehisc*):ti,ab,kw
#21 (wound* near/5 infect*):ti,ab,kw
#22 (wound* near/5 disrupt*):ti,ab,kw
#23 (wound next complication*):ti,ab,kw
#24 SSI:ti,ab,kw
#25 (or #13-#24)
#26 (and #12, #25) in Trials
